# Cordycepin inhibits cell senescence by ameliorating lysosomal dysfunction and inducing autophagy through the AMPK and mTOR–p70S6K pathway

**DOI:** 10.1002/2211-5463.13263

**Published:** 2021-08-27

**Authors:** Shi Qi Zuo, Can Li, Yi Lun Liu, Yue Hao Tan, Xing Wan, Tian Xu, Qiang Li, Li Wang, Yong Li Wu, Feng Mei Deng, Bin Tang

**Affiliations:** ^1^ School of Clinical Medicine Chengdu Medical College China; ^2^ School of Basic Medical Science Chengdu Medical College China; ^3^ Sichuan Clinical Research Center for Geriatrics Clinical Medical College and The First Affiliated Hospital of Chengdu Medical College China; ^4^ People's Hospital of Mingshan District Ya'an China; ^5^ Sichuan Second Hospital of Traditional Chinese Medicine Chengdu China

**Keywords:** AMPK signaling pathways, autophagy, cell senescence, cordycepin, lysosomal function, lysosomal protease

## Abstract

Cell senescence is closely related to autophagy. In this article, we identified a natural nucleoside analogue, cordycepin, that has the ability to significantly improve lysosomal function, enhance the activity of the lysosomal representative protease cathepsin B (CTSB), and promote the expression of the functional protein lysosomal‐associated membrane protein 2 (LAMP2) on the lysosomal membrane. Cordycepin then restores the damaged autophagy level of aging cells by activating the classic AMPK and mTOR–p70S6K signaling pathways, thus inhibiting cell senescence in an H_2_O_2_‐induced stress‐induced premature senescence (SIPS) cell model. This study provides new theoretical support for the further development of cordycepin and clinical antiaging drugs to inhibit cell senescence and suggests that the regulatory mechanisms of lysosomes in senescent cells should be considered when treating age‐related diseases.

## Graphical abstract

Here, we show that cordycepin enhances the expression of cathepsin B (CTSB) and lysosomal‐associated membrane protein 2 (LAMP2) and mediates the AMPK and mTOR‐p70S6K pathways to restore autophagy in aging cells.

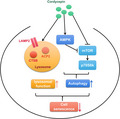

**Abbreviations**
ACP2: acid phosphatase 2
AMPK: adenosine 5’‐monophosphate‐activated protein kinase
C.C: compound C
CTSB: cathepsin B
LAMP2: lysosomal‐associated membrane protein 2
mTOR: mammalian target of rapamycin
p70S6K: p70S6 kinase
ROS: reactive oxygen species
SASP: senescence‐associated secretory phenotypes
SIPS: stress‐induced premature senescence

Cell senescence is an evolutionarily conserved state of stable replicative stagnation caused by stress. The accumulation of cell senescence leads to an increased burden on society and individuals [1]. Senescent cells exhibit the characteristics of large and flattened structural morphology, secretion of pro‐inflammatory factors and tissue remodeling factors, accumulation of lysosomal components, and impaired nuclear integrity [2]. Thus far, the mechanism of cell senescence has not been fully elucidated, and searching for active substances from nature to delay its process has become a major focus of current scientific research [3].

Cell senescence is closely related to autophagy. Autophagy can alleviate oxidative stress damage, eliminate damaged organelles, and protect cells [4]. AMPK and mTOR–p70S6K is a very common autophagy signaling pathway. Damage to autophagic flux and lysosomal dysfunction has been observed in senescent cells [5], and therefore, drug intervention for this target may be a prospective approach for combating cellular senescence. However, the development and application of targeted and specific antiaging clinical drugs are still lacking at present.

Cordycepin, also known as 3'‐deoxyadenosine, is a natural adenosine derivative. In addition to anti‐inflammatory [6], anti‐oxidation, and antitumor activity [7], cordycepin also induces autophagy by regulating the PKA/mTOR pathways or inhibiting cell senescence through the NRF2 and AMPK pathways [7]. Recently, Hueng *et al*. [8] found that cordycepin inhibits the migration of human glioblastoma cells by affecting lysosomal degradation and protein phosphatase activation. Other studies have revealed that cordycepin can restore the antioxidant status and decrease lipid peroxidation in aged rats [9]. Nonetheless, these studies, which investigated the effects of cordycepin on lysosome activity and aging, did not clearly explain the specific effects and underlying mechanisms.

In the current study, in order to clarify the relationship between cordycepin, autophagy, and cell senescence, we first chose to establish a stress‐induced premature senescence cell model to observe the effect of cordycepin on cell senescence. We then examined the specific regulation of cordycepin on autophagy flux and lysosomes. Our results verify that cordycepin is a promising anticell senescence drug that acts on the AMPK and mTOR–p70S6K signaling pathways, restores the lysosomal function of senescent cells, promotes autophagy levels, and delays cellular senescence.

## Materials and methods

### Cell culture and treatment of mouse fibroblasts

NIH3T3 mouse fibroblasts were cultured in Dulbecco’s modified high‐glucose medium with 10% fetal bovine serum at 37 °C and 5% CO_2_. The source of NIH3T3 cell lines is ATCC (CRL‐1658). To induce senescence [10], H_2_O_2_ (200, 400, and 800 µm) was added to the cells contained in a centrifuge tube and incubated at 37 °C. Every 5 min, the centrifuge tube was gently flipped once, for a total of nine times. NIH3T3 cells were then cultured in the prepared medium for 72 h in the presence of H_2_O_2_ and treated with different concentrations of cordycepin (0, 20, and 40 μm) for 48 h to prepare for the next tests. Each group had three replicates. Overall, the effect of cordycepin at a concentration of 40 μm was improved with 400 µm H_2_O_2_, and this concentration was used in subsequent experiments without special labeling.

### Enzyme‐linked immunosorbent assay (ELISA)

The concentrations of inflammatory cytokines IL‐1β, IL‐6, and TNF‐α in mice were determined by an ELISA kit (Beyotime, Shanghai, China, PI326; Shanghai Enzyme‐linked Biotechnology, Shanghai, China, ml063132 and ml002095). Each experiment was repeated three times in every group.

### SA‐β‐gal staining

The SA‐β‐gal activity level was detected by a SA‐β‐gal staining kit (Beyotime, C0602). Senescent cells express highly enzymatically active β‐galactosidase at pH 6.0, and dark blue products appear under the catalysis of this enzyme with X‐Gal as the substrate. Blue, highly expressed β‐galactosidase cells were observed under an optical microscope, and the statistical SA‐β‐gal‐positive ratio was determined. Representative images of three independent experiments for each group are presented.

### Western blot analysis

Cells were lysed using radioimmunoprecipitation assay buffer (Solarbio, Beijing, China, R0020). The proteins were quantified using the BCA kit (Beyotime, P0010). Protein samples were subjected to sodium dodecyl sulfate/polyacrylamide gel electrophoresis, transferred to PVDF membranes, and incubated for 90 min. The membranes were then blocked with 10% nonfat milk for 30 min and incubated overnight at 4 °C with antibodies against ATG7, ATG5 (Proteintech, Wuhan, China, 67341‐1, 66744‐1), p62, p53, p21, p16, LC3‐II/I, BECN1, mTOR, p‐mTOR, AMPK, p‐AMPK, p70S6K, p‐p70S6K (Cell Signaling Technology, Danvers, MA) 88588, 2524, 2946, 80772, 83506, 3738S, 4517, 5536, 5832, 5759, 9202, 9206), CTSB (Proteintech, 12216‐1), LAMP2 (Proteintech, 66301‐1), and GAPDH (Proteintech, 60004‐1). After incubation with secondary antibodies (Proteintech, SA00001‐1) for 90 min at 25 °C, the blots were reacted with SuperEnhanced ECL solution (GBCBIO Technologies Inc., Guangdong, China, G3308). Proteins were detected using the Image Lab software with a chemiluminescence apparatus (Bio‐Rad, Hercules, CA, USA). WB analysis for each group was repeated three times, and representative data are shown.

### Cell immunofluorescence

Cultured cells were fixed with 10% neutral formaldehyde in 24‐well plates for 10 min, then permeabilized with 0.5% Triton X‐100 for 15 min, and added goat serum to seal for 1 h. Sealed cells were incubated with the primary antibody at 4 °C overnight and then with the secondary antibody (1 : 500) for 1 h. Cell nuclei were stained with 4',6‐diamidino‐2‐phenylindole (DAPI, 1 : 10 000). During each operation interval, cells were washed three times with phosphate‐buffered saline (PBS) for 5 min each time. Staining intensity and images were observed using a fluorescence microscope (TCS SP5; Leica Microsystems, Wetzlar, Germany). Representative images from three replicates are shown.

### Cell reactive oxygen species (ROS)

A ROS detection kit was used with fluorescent probes. DCFH‐DA (Applygen, Beijing, China, C1300) with the appropriate volume of dilution was added to adherent culture cells and incubated for 20 min at 37 °C. The cells were collected and underwent analysis with a luciferase label analyzer. Three independent experiments were performed.

### LysoTracker Red DND‐99 analysis

The medium was removed from the dish, and prewarmed (37 °C) LysoTracker Red (Beyotime, C1046) probe‐containing medium prepared in advance was added when the cells reached the desired confluence. The cells were incubated for 30 min at 37 °C. The loading solution was replaced with fresh medium, and lysosomes were observed using a fluorescence microscope. We observed at least 50 cells in each group and selected the representative images from three independent experiments.

### Lysosomal acid phosphatase activity detection

The cells were lysed with lysis buffer (Beyotime; P0013J), and after centrifugation, the supernatant was obtained for phosphatase activity detection using an acid phosphatase assay kit (Beyotime, P0326). The test consisted of a control along with 4, 8, 16, 24, 32, and 40 μL of standard product and 40 μL of sample. After directly incubating for 5–10 min at 37°C, 160 μL stop solution was added to terminate the reaction. Absorbance was measured at 405 nm, and acid phosphatase activity was calculated according to the definition of enzymatic activity.

### Statistical analysis

spss 13.0 software and graphpad prism 5 (GraphPad Software Inc., La Jolla, CA, USA) were used for statistical analysis, and the data are expressed as the mean ± standard deviation using three biological replicates. Student's *t*‐test and univariate ANOVA were used to determine the statistical significance. The asterisk represents statistical significance (*P* < 0.05).

## Results

### Cordycepin delayed cell senescence

**Fig. 1:**
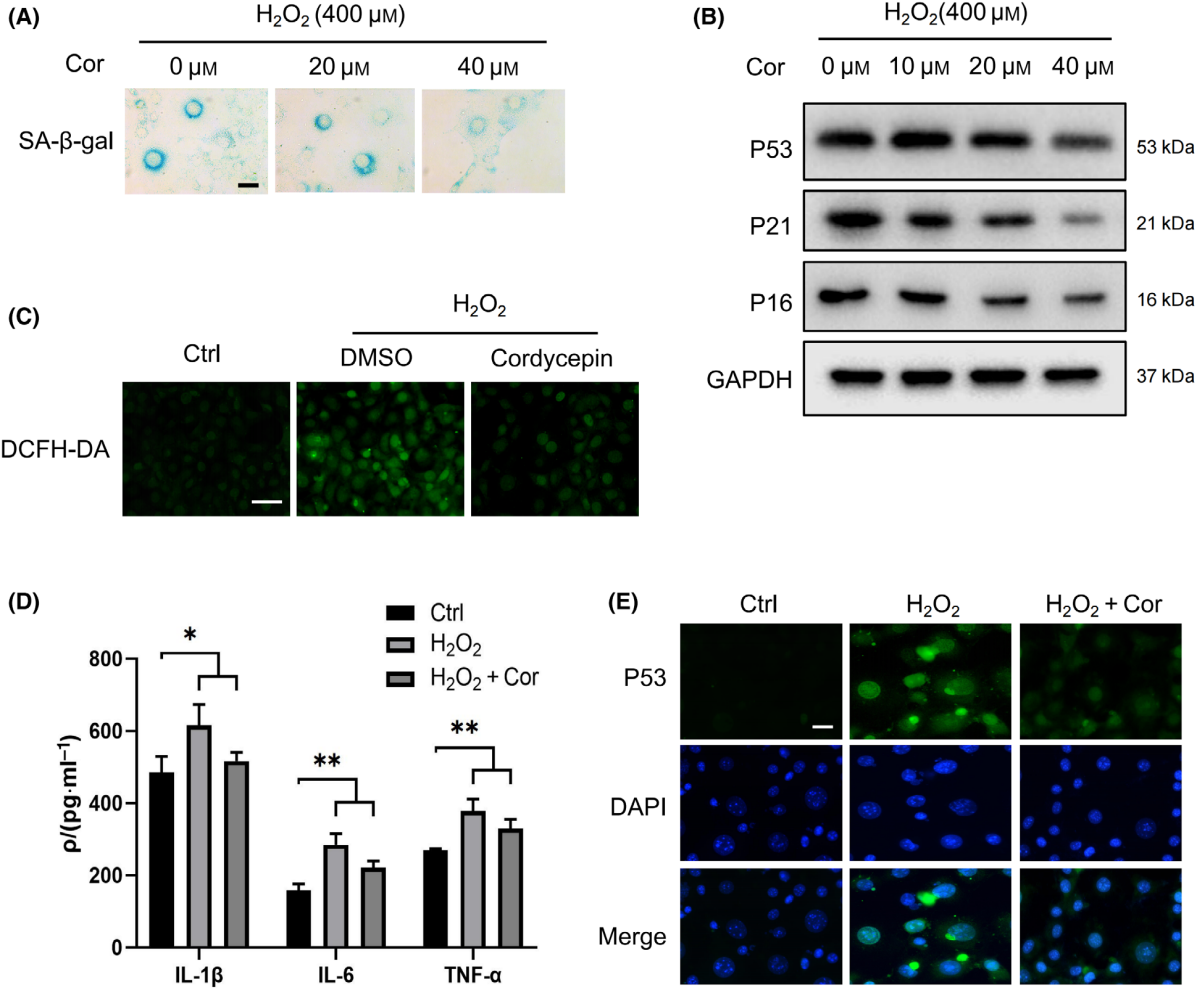
Cordycepin delays cell senescence. (A) NIH3T3 cells were stained using a SA‐β‐gal assay kit after treatment with 400 μm H_2_O_2_ for 72 h, and representative images were selected from three independent tests. Scale bars = 20 μm. (B) Western blot (WB) image of aging marker proteins P53, P21, and P16 in NIH3T3 cells after hydrogen peroxide treatment at different concentrations. (C) DCFH‐DA fluorescence was used to measure the amounts of intracellular reactive oxygen species (ROS). Scale bars = 100 μm. (D) ELISA was used to detect different levels of pro‐inflammatory factors in the control group, senescence group, and cordycepin treatment group. (E) The immunofluorescence images show the changes in the senescence marker protein P53 in the control group, the senescence group, and the cordycepin treatment group. Scale bars = 50 μm. Representative images of three independent experiments are presented. Data were analyzed using Student’s *t*‐test and are presented as the mean ± SD. *P < 0.05, **P < 0.01. The following concentrations were used in the treatments depicted in C, D, and E: H_2_O_2_ (400 μm) and cordycepin (40 μm).

To study the effect of cordycepin on cell senescence, we established an H_2_O_2_ stress‐induced premature senescence (SIPS) cell model [10]. Mouse fibroblasts (NIH3T3) were treated with different concentrations of hydrogen peroxide (200, 400, and 800 μm) and cultured for 3 days after treatment. There was a tremendous increase in SA‐β‐gal activity (Fig. S1A), and the protein levels of aging markers P53, P21, P16 also increased (Fig. S1B). We next carried out the detection of reactive oxygen species (ROS) using a DCFH‐DA fluorescent probe, and the result showed that hydrogen peroxide treatment increased intracellular ROS levels (Fig. 1C). Compared to the uninduced cells, there was a marked increase in the levels of IL‐1β, IL‐6, and TNF‐α, which is known as the senescence‐associated secretory phenotype (SASP) in senescent cells (Fig. 1D). These results suggest that using 400 µm hydrogen peroxide can successfully induce cell senescence.

To determine the effect of cordycepin on cell senescence, NIH3T3 cells were treated with cordycepin using a 0, 20, 40 μm concentration gradient for 2 days. It was observed in the aging group that the SA‐β‐gal‐positive staining rate and SASP level both decreased at higher concentrations (40 μm) of cordycepin (Fig. 1A,D). Western blot showed decreased protein levels of aging markers P53, P16, and P21 (Fig. 1B), and the immunofluorescence assay displayed decreased protein expression of P53 (Fig. 1E). Furthermore, the amount of intracellular ROS also decreased (Fig. 1C). These results suggest that cordycepin may increase the antioxidant capacity of cells and delay cell senescence.

### Cordycepin promoted autophagy in senescent cells

**Fig. 2:**
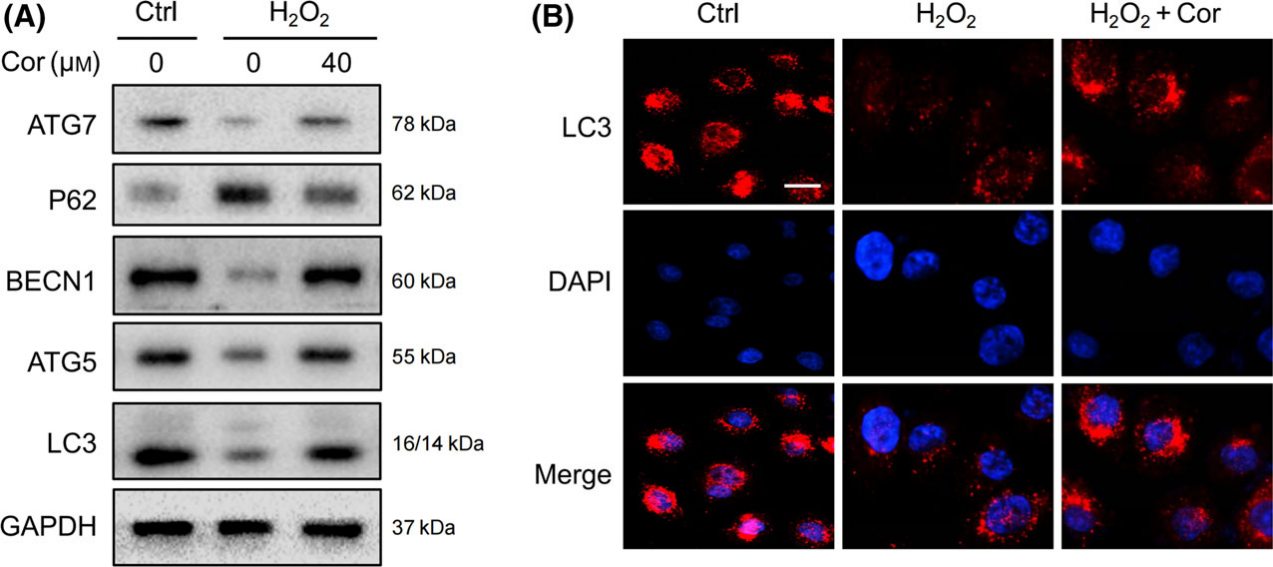
Cordycepin promotes autophagy in senescent cells. (A) Western blot analysis of autophagy‐related protein levels in cordycepin‐ and H_2_O_2_‐treated NIH3T3 cells. (B) Immunofluorescence images show the changes in LC3‐positive staining distribution in the control group, the senescent group, and the cordycepin group. The representative images of three independent experiments are presented. Scale bars = 20 μm.

It has been illustrated that there is damage to the autophagic flux in aging cells, and restoring the autophagy level can alleviate cell senescence to some extent [5]. Upon induction of cell senescence, we found that the autophagy level decreased, cell‐related autophagy proteins ATG5, ATG7, and BECN1 expression decreased, and autophagy substrate P62 expression increased (Fig. 2A). To confirm whether cordycepin delayed cellular senescence by affecting autophagy, we examined changes in autophagy levels within the two groups, 24 h after the addition of cordycepin. The results of western blotting (WB) showed that the LC3‐II/I ratio and expression of ATG5 and ATG7 increased, while the expression of the autophagy substrate, P62, decreased, following cordycepin treatment (Fig. 2A). ATG5 and ATG7 are long believed to be essential for autophagy, and the conversion of LC3 is considered to be a reliable marker of autophagy [11]. Immunofluorescence results also showed an increased number of aggregated LC3 spots after cordycepin treatment (Fig. 2B). Therefore, cordycepin promotes autophagy, which is beneficial for senescent NIH3T3 cells.

### Cordycepin delayed cellular senescence by affecting AMPK and mTOR–p70S6K pathway

AMPK is a late‐stage endosomal/lysosome‐resident protein and can be activated on the lysosomal membrane [12]. Mammalian target of rapamycin (mTOR) is a crucial regulator of autophagy initiation that includes two complexes, mTORC1 and mTORC2. It has been reported that mTORC1 promotes reliable signaling for the longevity of different organisms, and deletion of its downstream substrate p70S6 kinase (p70S6K) results in increased lifespan in female mice [13]. We detected the effect of the AMPK and mTOR–p70S6K signaling pathway on cell senescence by treating senescent cells with metformin. Past studies have reported that metformin treatment increases AMPK signaling activity [14]. We found that AMPK signaling and increased LC3 conversion altered the expression of p70S6K, p53, p21, and p16 (Fig. S2A,B), which was concomitant with increasing SASP factor levels (Fig. S2C). Fluorescence microscopy showed that the number of LC3 dots increased after metformin treatment, similar to that of cordycepin (Fig. S2D). These results reveal that the AMPK and mTOR–p70S6K signaling pathways promote autophagy and reduce cell senescence.

**Fig. 3:**
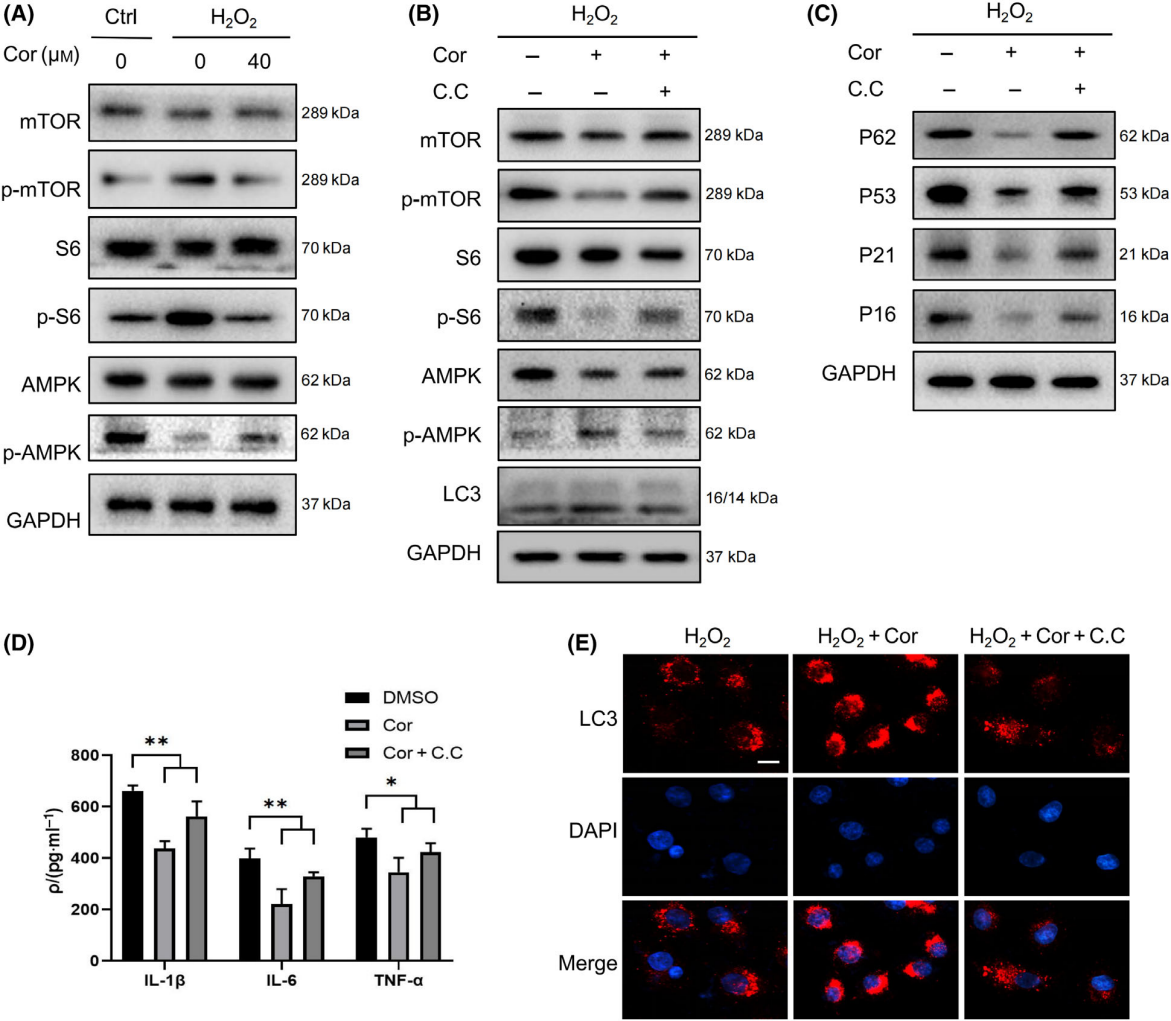
Cordycepin affects AMPK and mTOR–p70S6K autophagy access to delay cellular senescence. (A) WB analysis of mammalian target of rapamycin (mTOR), p70S6 kinase (p70S6K), p‐p70S6K, adenosine 5′‐monophosphate‐activated protein kinase (AMPK), and p‐AMPK expression in control and senescent fibroblasts after treatment with 40 μm cordycepin for 48 h. (B) WB images of autophagy, mTOR, AMPK, and p70S6K protein levels in senescent cells treated with cordycepin and compound C (C.C). (C) WB images of autophagy and aging protein levels in senescent cells treated with cordycepin and C.C. (D) ELISA image of expression of different senescence‐associated secretory phenotypes (SASP) under treatment with 400 μm hydrogen peroxide. Data were analyzed using Student’s *t*‐test and are presented as the mean ± SD. *P < 0.05, **P < 0.01. (E) Immunofluorescence shows the changes in LC3 fluorescence staining in the senescent group and after the addition of cordycepin and C.C, and the representative images of three independent experiments are shown. Scale bars = 20 μm.

Furthermore, to explore the potential mechanism used by cordycepin to regulate autophagy, we examined the effect of cordycepin on the AMPK and mTOR–p70S6K signaling pathway. WB showed that treatment of NIH3T3 cells with cordycepin reduced p‐mTOR and p70S6K phosphorylation levels and significantly increased levels of phosphorylated AMPK (Fig. 3A). These results substantiate our claim that cordycepin can act on this signaling pathway.

Egan *et al*. [15] found that loss of AMPK results in aberrant accumulation of the autophagy adaptor, p62, and defective mitophagy. To clarify whether the effect of cordycepin on autophagy and cell senescence depends on the AMPK and mTOR–p70S6K signaling pathway, we treated NIH3T3 cells with an AMPK inhibitor, compound C (C.C), and observed whether cordycepin can alleviate cell senescence after inhibiting the AMPK target. Senescence and autophagy marker proteins were determined by western blot. p‐AMPK expression and LC3‐II/I ratio obviously decreased after C.C treatment, with an increase in p‐mTOR, p‐p70S6K, P62, senescence protein, SASP levels, and LC3 dots (Fig. 3B–E). In general, cordycepin can delay cellular aging through the AMPK and mTOR–p70S6K pathway.

### Cordycepin ameliorated lysosomal dysfunction in senescent cells

**Fig. 4:**
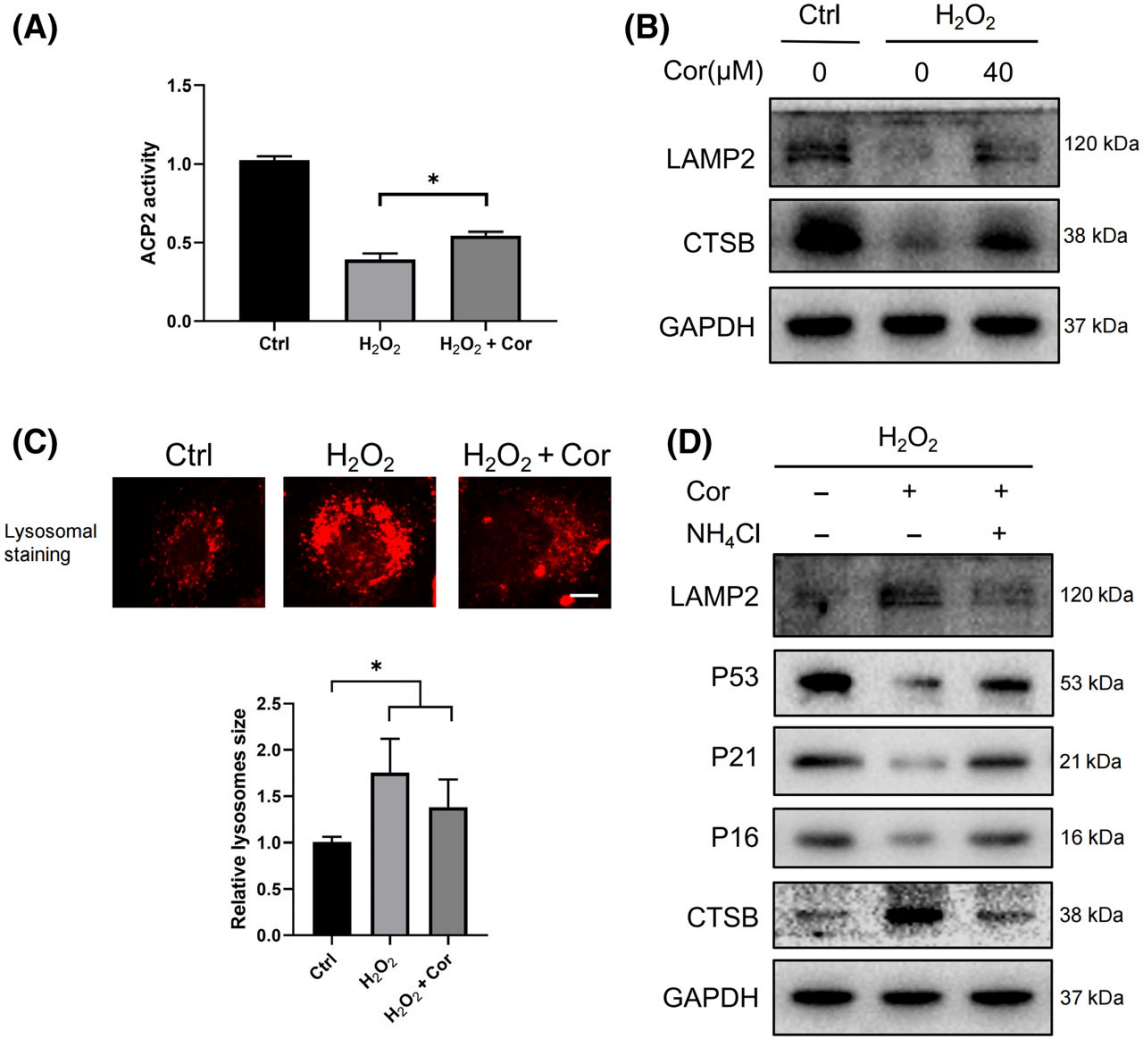
Cordycepin ameliorated lysosomal dysfunction in senescent cells. (A) Acid phosphatase activity was detected after H_2_O_2_ and cordycepin treatment. The data were analyzed using Student’s *t*‐test and are shown as the mean ± SD. *P < 0.05. (B) The control group and senescent fibroblasts were treated with 40 μm cordycepin for 48 h, and the WB indicates the expression of lysosomal‐associated membrane protein 2 (LAMP2) and representative enzyme cathepsin B (CTSB) on the lysosomal membrane. (C) The lysosomal status was probed with a LysoTracker Red DND‐99 kit after senescent cells were treated with cordycepin, and representative images of three independent replicates are shown. Scale bars = 10 μm. (D) WB detected the expression of senescence‐related protein as well as LAMP2 and CTSB after treatment with cordycepin alone and with NH_4_Cl. Data were analyzed using Student’s *t*‐test and are presented as the means ± SD. *P < 0.05, **P < 0.01.

Lysosomes not only are the degradation centers of the autophagy pathway but have also been found in recent years to act as regulatory points for complex intracellular signaling networks [16]. Earlier, we demonstrated the possible regulatory mechanism used by cordycepin on cell senescence and autophagy. We speculated that cordycepin may also play a role in regulating lysosomal function because both AMPK and mTOR can be activated on the lysosomal membrane. Acid phosphatase is a marker enzyme in lysosomes, and our results indicate that cordycepin restored the depressed lysosomal acid phosphatase 2 (ACP2) activity in senescent cells (Fig. 4A). We used two proteins, CTSB and LAMP2, to detect lysosomal function. CTSB is a representative protease in lysosomes, and the expression of LAMP2 is proportional to autophagy activity; thus, it contributes to the clearance of damaged proteins [17]. The WB data suggest that cordycepin promoted CTSB and LAMP2 expression in senescent cells (Fig. 4B). Lysosomal staining shows an increase in lysosomal structure in senescent cells, and cordycepin diminished this change (Fig. 4C). To determine the positive effects on autophagy and cell senescence, ambroxol, a lysosome maturation promoter, was used to treat control group and senescent group cells. It was obvious that after adding ambroxol, the expression of cell senescence marker proteins decreased, and the expression of IL‐1β, IL‐6, and TNF‐α, which are characteristic factors of SASP, decreased (Fig. S3A,B). As mentioned, promoting lysosomal maturation enhanced autophagy and decreased cell senescence levels.

To clarify whether cordycepin plays a role in regulating lysosomes, the lysosomal inhibitor NH_4_Cl was used to treat cordycepin‐treated senescent cells. WB exhibited decreased expression of lysosomal representative proteases CTSB and LAMP2 on the membrane after treatment with lysosomal inhibitors, with an increase in aging proteins (Fig. 4D). These results confirm that cordycepin can alleviate lysosomal dysfunction and slow the process of cell senescence.

## Discussion

After studying the role and mechanism of cordycepin in regulating autophagy and cell senescence, we discovered that cordycepin is beneficial for improving lysosomal function and acting on the AMPK and mTOR–p70S6K signaling pathway to promote autophagy and delay cell senescence.

The progressive development of aging is a major risk factor for many age‐related diseases. The United Nations report on aging noted that the aging of the world's population in the 21st century has the characteristic of being ‘universal, deep, sustained, unreversible’ [18]. Because of the increasing burden on society, it is now being treated as a global medical problem.

Cell senescence and autophagy are closely connected. Previous studies have focused on the effects of autophagy structure and activity, the molecular mechanisms of autophagy, and the genetic regulation of cell senescence during autophagy [19]. However, because of the differences in treatment factors, such as different drug properties, reaction times, and cell activity, the relationship between autophagy and cellular senescence remains inconclusive. In Drosophila studies, a shorter lifespan was measured after decreased expression of Atg genes, which are necessary for autophagy [20]. Our experiments also showed that promoting autophagy to degrade cell components will delay cell senescence. Cordycepin‐treated mouse fibroblasts exhibited increased autophagy and decreased cellular senescence, which also provides new evidence that drugs can be used to delay cell senescence by regulating autophagy.

Cordycepin is a metabolic product isolated from cultures of the insect‐pathogenic fungus *Cordyceps militaris* and was originally extracted from a *C. militaris* culture that was grown from the conidia by Cunningham *et al*. [21]. Cordycepin has strong anticancer, antioxidant, and anti‐inflammatory effects and also acts as a natural nucleoside analogue. The role of cordycepin in the liver, kidney, cardiovascular, and immune systems and with ischemia‐reperfusion injury has also been discovered in recent years [22, 23]. A previous study on the association between adenosine 5′‐monophosphate‐activated protein kinase (AMPK) and cordycepin provided evidence that cordycepin acts on the γ1 subunits of AMPK, which can regulate lipid metabolism in HepG2 cancer cells [24]. Moreover, cordycepin can act directly on the AMPK α1 and γ1 subunits and activate AMPK without altering the AMP/ATP ratio or upstream kinase activity of AMPK [7]. AMPK is a major regulator of metabolism and is a heterotrimer protein that can be activated on lysosomes [12]. Previous studies have shown that the increased intracellular ratio of ADP:ATP and AMP:ATP is the main mechanism responsible for AMPK activation [25]. In addition, a portion of AMPK metabolic regulation is mediated by mTORC1, by inhibiting its phosphorylation and results in promoting autophagy [26]. Selman and others reported that deletion of the mTOR downstream kinase p70S6 kinase (p70S6k) can prolong the lifespan of female mice [13].

The current research on the effect of cordycepin on AMPK is mainly focused on the regulation of AMPK’s specific structure, and studies of the molecular mechanism have not been reported in detail yet [27]. The complex signaling pathway underlying the mechanism of action of cordycepin on autophagy remains unclear. Previous studies have shown that the antagonism between AMPK and Akt is bidirectional, while C.C can induce the phosphorylation of Akt1. Therefore, the activation of AMPK by cordycepin may be a secondary effect of the inhibition of Akt signaling, which is blocked by C.C. However, the specific mechanism is still unclear and needs further confirmation [28]. Hardie *et al*. [29] also explained that C.C is a relatively nonselective inhibitor, and therefore, more inhibitors that are specific need to be identified. In the current study, we observed that cordycepin enhanced the levels of phospho‐AMPK and decreased the expression of mTOR with its downstream substrate p70S6K, subsequently enhancing autophagy in senescent cells. Interestingly, accumulating evidence shows that the degradation of autolysosomal products reactivates mTOR, thus attenuating autophagy and generating protolysosomal tubules and vesicles that extrude from autolysosomes. Lastly, they mature into functional lysosomes, thereby restoring the full complement of lysosomes [30]. The research on the detailed regulatory mechanisms of cordycepin that govern autophagy remains an ongoing exploration, and it is worthwhile to devote additional effort to this.

Autophagy is an evolutionarily conserved process through which damaged organelles and proteins are engulfed mainly by autophagosomes, which later fuse with lysosomes to form autolysosomes; thus, proteins are degraded in the autophagosome [31]. The lysosome is an acidic organelle that contains multiple hydrolases. During the autophagosome and lysosome fusion, an important functional protein on the lysosomal membrane, LAMP2, plays an essential role [32]. Stabilization of LAMP2 in senescent cells and prevention of its age‐dependent decline in expression may delay senescence [33]. In our experiments, cordycepin promoted the expression of LAMP2, acid phosphatase, and CTSB in lysosomes. In previous studies, disorders of lysosomal function had been observed in models of stress‐induced premature aging, and lysosomal dysfunction occurred comparatively late [5]. Our results showed that cordycepin can reverse the damaged lysosomal function in senescent cells, but we cannot clearly confirm the mechanism used by cordycepin to regulate lysosomal function. Also, studies on the effect and association of cordycepin with lysosomes have yet to be identified.

A recent article revealed that cordycepin affected lysosomal degradation and protein phosphatase activation, with an inhibitory effect on the migration of tumor cells [8]. Past research has shown that AMPK and mTOR can be located and activated on lysosomal membranes. Consistent with previous studies, other evidence also proved that AMPK and mTOR share common activation pathways, such as lysosomal v‐ATPase‐regulator complex, an initial energy stress sensor that regulates catabolism [12]. However, we did not further explore whether the effect of cordycepin on lysosomes depends on this particular AMPK and mTOR–p70S6K signaling pathway, or whether cordycepin signals lysosomes to recognize damaged organelles and protein and the direction and rate of lysosome movement. For refinement, these ideas still require future research and discovery.

Progressive mitochondrial dysfunction can lead to the accumulation of ROS. The results of our study also indicated that cordycepin can decrease the superoxide anion level in aging cells. During autophagy, damaged mitochondria are degraded through lysosomes to maintain cellular homeostasis. Increasing evidence provides support for the theory that there are possible interactions between the two important cellular metabolic and homeostasis regulators, mitochondria and lysosomes. For example, when mitochondrial dysfunction occurs, there are sustained adverse effects on lysosomes in different types of cells [34]. It has also been found that a defective mitochondrial respiratory chain leads to an AMPK signaling pathway malfunction, resulting in impaired lysosomal catabolism [35]. The mitochondrial–lysosome axis is widely involved in many diseases [36], and therefore, the specific mechanism that regulates the response of cordycepin against oxidation and lysosomes can be further explored.

The current study confirms for the first time that cordycepin potentially restores lysosomal dysfunction, promoting lysosomal protease activity of CTSB and increasing the expression of LAMP2, which mediates autophagy and lysosomal fusion on the lysosomal membrane. Moreover, cordycepin also activates phosphorylated AMPK to inhibit the expression of mTOR and p70S6K in a SIPS cell model, thus increasing autophagy and alleviating cell senescence. We also provide novel insights into alternative targets and the clinical application of cordycepin. Furthermore, the specific mechanism of cordycepin on autophagy and lysosomal function and the effect of cordycepin on cell senescence and senescence‐related diseases will be further elucidated in future research.

## Conflict of interest

The authors declare no conflict of interest.

## Author contributions

BT and FMD designed and conducted the project. SQZ, YHT, XW, TX, QL, LW, and YLW analyzed and interpreted the data. SQZ, CL, and YLL wrote the paper. FMD and BT supervised the project and modified the manuscript.

## Supporting information

**Fig. S1**. The H2O2 ‐induced cell oxidative stress aging model was established.
**Fig. S2**. The AMPK agonist metformin altered the AMPK and mTOR–p70S6K pathway to delay cell senescence.
**Fig. S3**. Ambroxol promoted lysosomal function and delayed cell senescence.

## Data Availability

The data that support the findings of this study are available from the corresponding author upon reasonable request.
