# Supplementary material for: Cordycepin inhibits cell senescence by ameliorating lysosomal dysfunction and inducing autophagy through the AMPK and mTOR–p70S6K pathway
Source: FEBS Open Bio. 2021 Aug 27;11(10):2705–14. doi: 10.1002/2211-5463.13263 (PMC8487049; doi:10.1002/2211-5463.13263)
Supplement: Supplementary file 1 — Fig. S1. The H2O2 ‐induced cell oxidative stress aging model was established. Fig. S2. The AMPK agonist metformin altered the AMPK and mTOR–p70S6K pathway to delay cell senescence. Fig. S3. Ambroxol promoted lysosomal function and delayed cell senescence. [file FEB4-11-2705-s001.pdf]

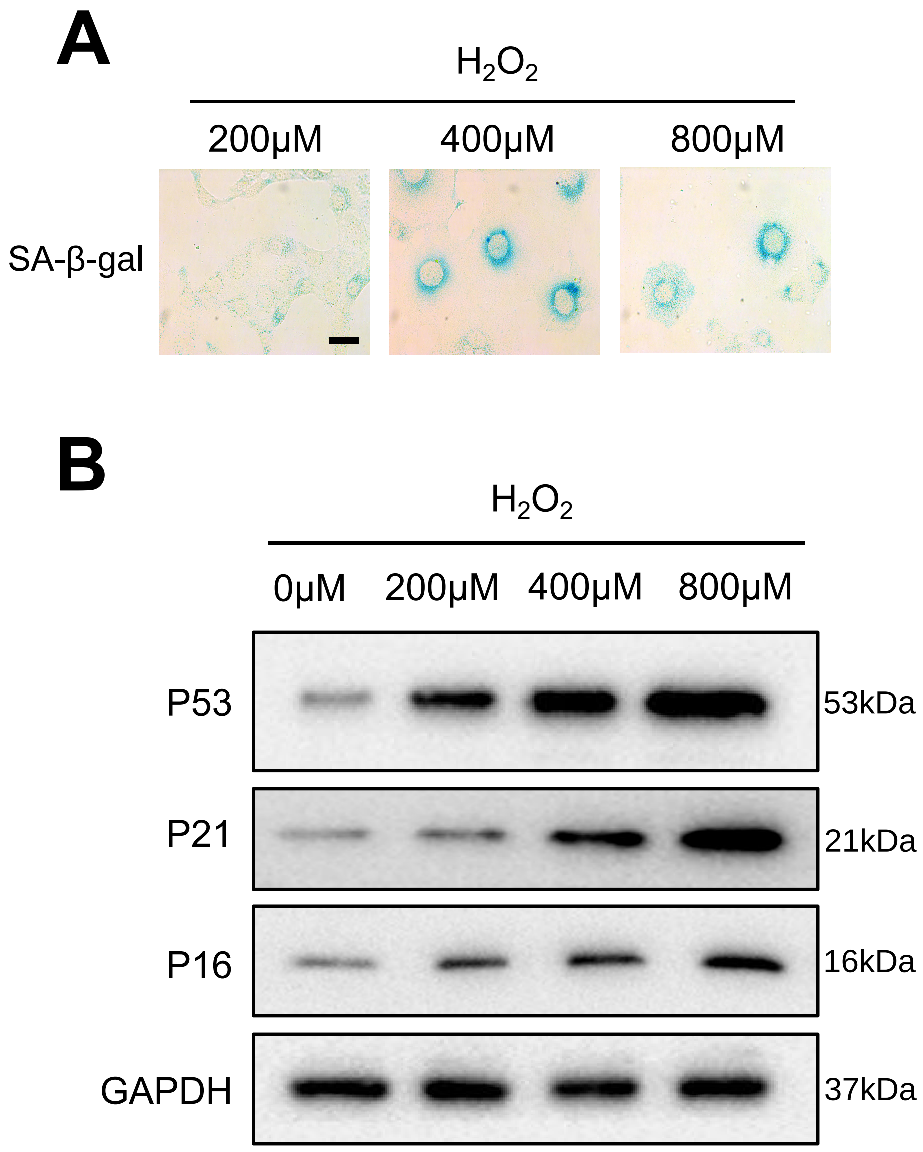

Supplementary Figure 1. The H_2O_2-induced cell oxidative stress aging model was established. (A) The cells were treated with different concentrations of hydrogen peroxide for 72 h before SA-β-gal staining. The experiment was repeated three times, and representative images were selected. Scale bars = 20 μm. **(B)** WB image of aging marker protein after treatment of aging cells with different concentrations of cordycepin.

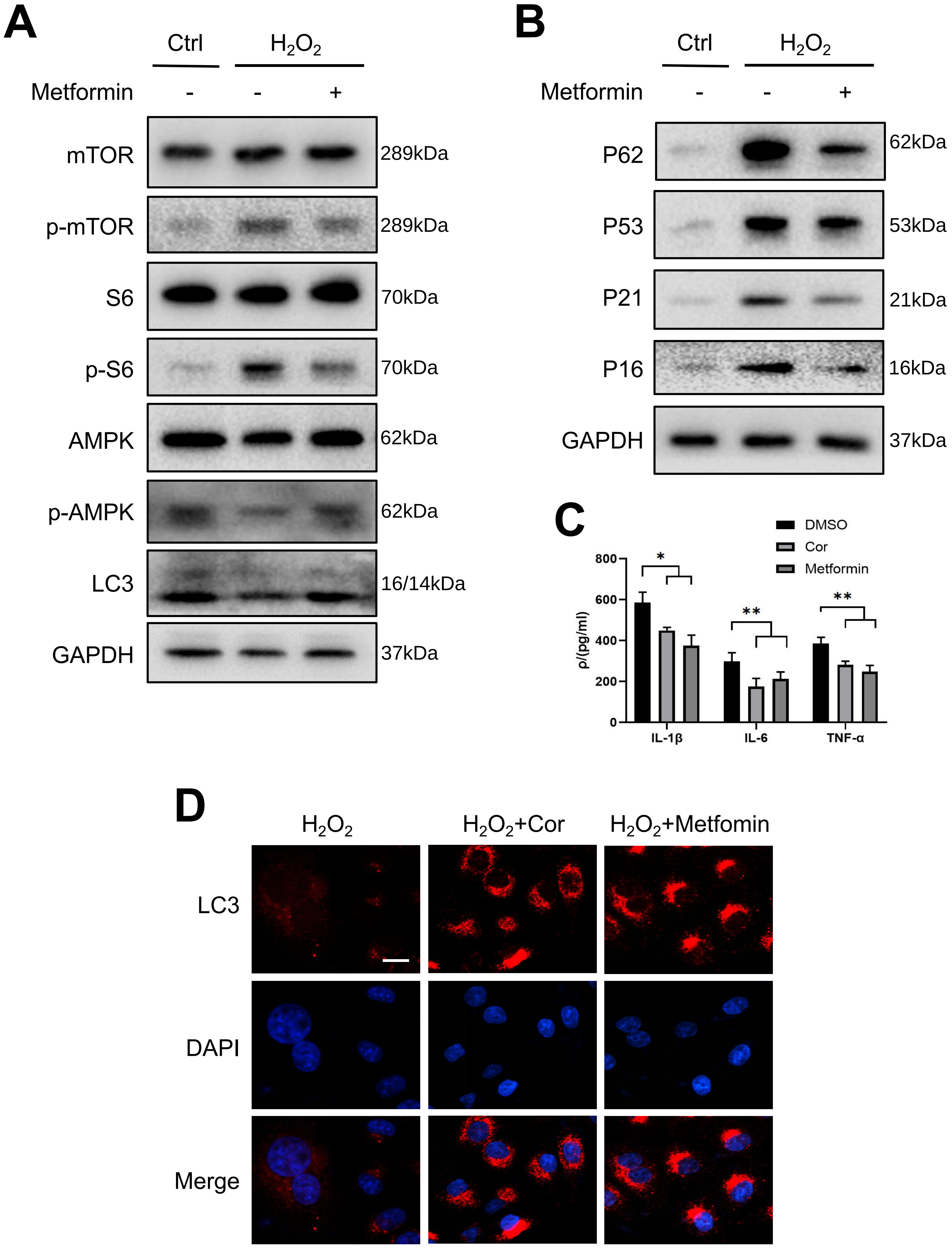

Supplementary Figure 2. The AMPK agonist metformin altered the AMPK and mTOR-p70S6K pathway to delay cell senescence. (A) Control and senescent cells were treated with metformin. WB was used to determine autophagy level, and expression of mTOR, AMPK, and p70S6K and their phosphorylation levels. **(B)** WB was used to determine autophagy and senescence levels. **(C)** ELISA was used to detect the levels of proinflammatory factors TNF-α, IL-6, and IL-1β in the control group, and the cordycepin and metformin treatment groups after adding hydrogen peroxide. The data are shown as the mean±SEM. $*P < 0.05$, $**P < 0.01$. **(D)** The changes in the LC3 content in senescent cells treated with cordycepin and metformin were compared in fluorescent images. Scale bars = 20 μm.

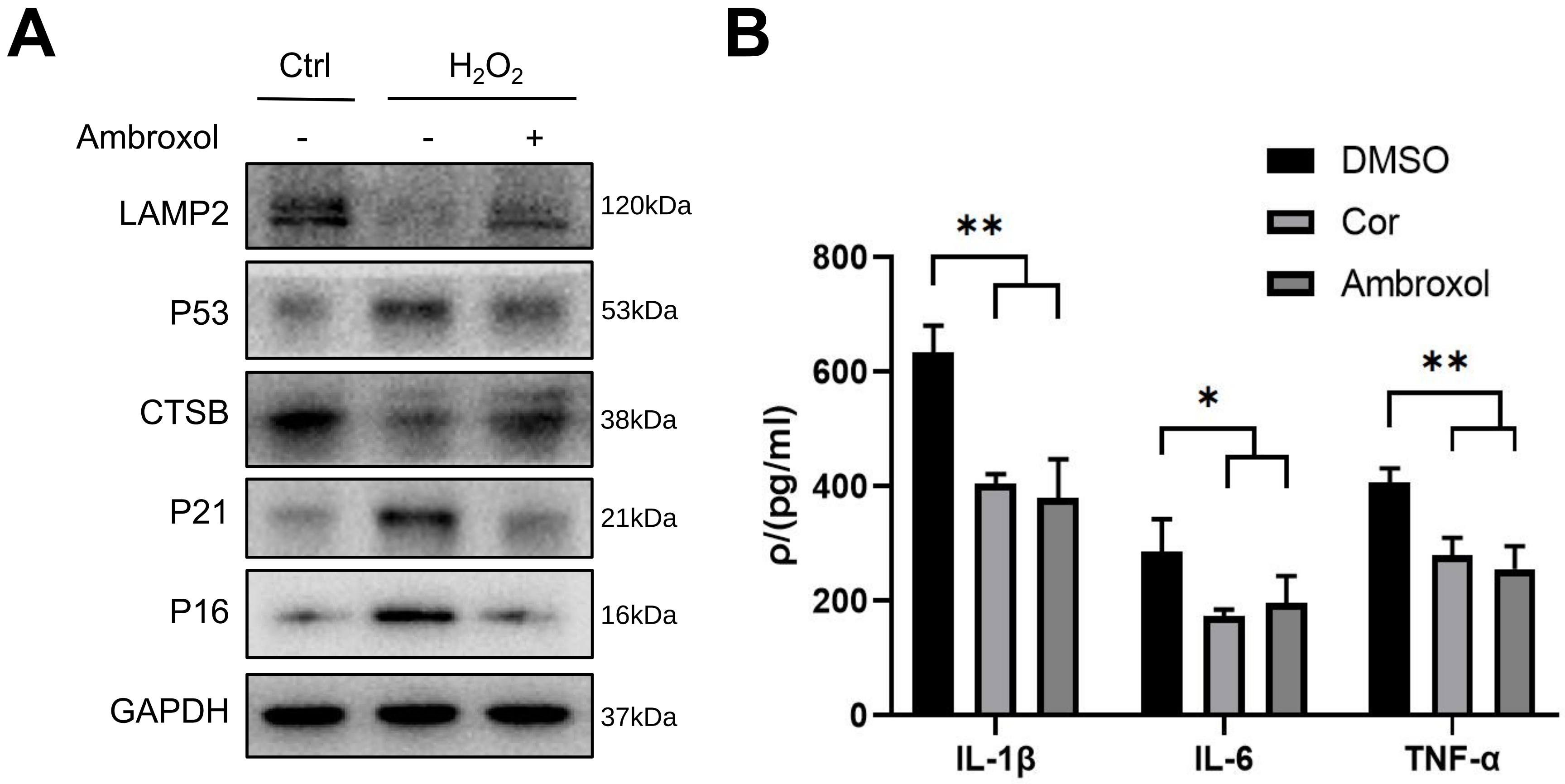

Supplementary Figure 3. Ambroxol promoted lysosomal function and delayed cell senescence. (A) Adding ambroxol promoted lysosomal maturation, and WB analysis indicates the levels of senescence- and lysosomal-related enzymes. **(B)** ELISA was used to detect the expression in senescent cells of three SASP factors for the control group, and cordycepin- and ambroxol-treated groups. The data are shown as the mean ± SEM. *$P < 0.05$, **$P < 0.01$.
